# Immunotherapy for lung adenocarcinoma patients with bone metastases: who really needs it

**DOI:** 10.3389/fimmu.2024.1457916

**Published:** 2024-12-13

**Authors:** Zhangheng Huang, Yuexin Tong, Lujian Zhu, Binbin Yang, Kai Chen, Peiling Dai

**Affiliations:** ^1^ Department of Orthopaedics (Spine Surgery), The First Affiliated Hospital of Wenzhou Medical University, Wenzhou, Zhejiang, China; ^2^ Department of Spine Surgery, The Second Affiliated Hospital of Wenzhou Medical University, Wenzhou, Zhejiang, China; ^3^ Department of Infectious Diseases, Affiliated Jinhua Hospital, Zhejiang University School of Medicine, Jinhua, Zhejiang, China; ^4^ Department of Reproductive Medicine, The First Affiliated Hospital of Wenzhou Medical University, Wenzhou, Zhejiang, China; ^5^ Department of Cardiovascular and Thoracic Surgery, The Second Affiliated Hospital of Wenzhou Medical University, Wenzhou, Zhejiang, China; ^6^ Department of Radiotherapy, The First Affiliated Hospital of Wenzhou Medical University, Wenzhou, Zhejiang, China

**Keywords:** immunotherapy, lung cancer, bone metastases, SEER, prognosis

## Abstract

**Background:**

Lung adenocarcinoma patients are often found to have developed bone metastases at the time of initial diagnosis. With the continuous development of technology, we have successfully entered the era of immunotherapy. This study aimed to determine the efficacy of immunotherapy in lung adenocarcinoma patients with bone metastases (LABM) through a multicenter retrospective analysis and to develop a novel tool to identify the population that could benefit most from immunotherapy.

**Methods:**

To assess the impact of immunotherapy on LABM in terms of overall survival, we used analytical tools such as Kaplan-Meier analysis, Log-ranch test, and propensity score matching (PSM) method. A predictive model for constructing overall survival was constructed using Cox regression modeling. Based on this, we developed a risk classification system depicting Kaplan-Meier curves for subgroup analysis to determine the optimal beneficiary population for immunotherapy in different risk subgroups.

**Results:**

A total of 20073 eligible patients were enrolled in this study, of whom 8010 did not receive immunotherapy, while 12063 patients received immunotherapy. After 1:1 PSM, 15848 patients were successfully coordinated, yielding a balanced cohort. Kaplan-Meier survival curves showed significantly enhanced overall survival (P < 0.001) in patients who received immunotherapy compared to those who did not. The results of Cox regression analyses showed that age, race, sex, primary site, immunotherapy, surgery, chemotherapy, brain metastasis, liver metastasis, lung metastasis, and marital status were independent prognostic factors. The area under the curve for all three cohorts was close to 0.7, indicating that the model was well-discriminating. The calibration curves further proved that the model had a high predictive accuracy. Decision curve analysis demonstrated that the model could achieve a high net clinical benefit. The risk classification system developed based on the model successfully screened the best beneficiary population for immunotherapy.

**Conclusion:**

This study provides convincing evidence that immunotherapy provides a significant survival advantage for LABM. Secondly, the clinical tools constructed in this study can help clinicians identify the optimal population to benefit from immunotherapy in LABM, thus enabling precise treatment and avoiding the waste of medical resources and over-treatment of patients.

## Introduction

Lung cancer is the most common malignant tumor in the world, with approximately 1.8 million new cases of lung cancer diagnosed worldwide, including 1.6 million deaths (1). Over 85% of lung cancer patients are diagnosed with non-small cell lung cancer, with lung adenocarcinoma being the most common histologic type (2). Early symptoms of lung cancer are atypical, which makes early diagnosis particularly difficult. When patients develop more obvious clinical symptoms such as hemoptysis, chest pain, and chest tightness, the disease may have progressed to an advanced stage, often accompanied by distant metastases (3). Among them, bone is the most common site of metastasis in lung cancer patients, and about 30%-40% of patients have bone metastasis (4). Bone metastasis not only has a significant impact on the quality of life of patients but also significantly reduces the survival time of patients. Among patients with bone metastasis of lung cancer, 50.3% are adenocarcinoma, and the most common sites of bone metastasis are the spine and trunk bone (5). Therefore, we may need to pay more attention to lung adenocarcinoma patients with bone metastases (LABM).

Surgery is considered an effective treatment for early-stage lung cancer, but it is usually not considered the preferred treatment option for patients with advanced lung cancer. This is because even if surgery is chosen, postoperative recurrence and distant metastasis remain unavoidable (6). Since the main treatment goals of these patients are to relieve pain, improve their quality of life, and prolong survival, systemic therapy is usually chosen. In this regard, the role of immunotherapy in lung cancer patients has received increasing attention, especially with the significant advances in the use of immune checkpoint inhibitors (7). Immunotherapy works by enhancing or modifying the patient’s immune system so that it can recognize and attack cancer cells (8). Compared to traditional treatments (e.g., radiotherapy, chemotherapy), immunotherapy is more specific in its targeting, providing long-term control and fewer side effects (9). In lung cancer patients with bone metastases, immunotherapy may work by modulating the tumor microenvironment and activating immune cells (10). Previous studies have shown that immunotherapy significantly prolongs progression-free survival and overall survival (OS) in patients with non-small cell lung cancer, bringing hope to patients with advanced lung cancer (11–13). In 2015, immunotherapy was officially approved as a standard treatment option for patients with advanced non-small cell lung cancer (11). Since bones have a unique immune microenvironment different from other organs, the efficacy of immunotherapy may be compromised for lung cancer patients with bone metastases (14). Currently, few studies have been conducted to report the efficacy of immunotherapy in LABM, and most of them are studies with limited sample sizes. Therefore, the exact mechanism and efficacy of immunotherapy in LABM need to be further investigated.

Although immunotherapy has been reported to provide survival benefits for lung cancer patients with bone metastases, it is undeniable that not all lung cancer patients with bone metastases benefit from it. Therefore, this study aimed to retrospectively analyze data from LABM from a multicenter medical institution to verify the efficacy of immunotherapy with LABM. At the same time, a practical mortality risk classification system was developed on this basis, which was further validated with patient data from external medical institutions. By using the mortality risk classification system, we can identify the largest beneficiary population of immunotherapy among LABM, which provides the basis for personalized and precise treatment for patients.

## Methods

### Patient cohort

Data for this study were obtained in part from the Surveillance, Epidemiology, and End Results (SEER) database, which encompasses cancer-related demographic data from 17 U.S. cancer registries covering approximately 30% of the U.S. population. The database provides comprehensive information on patient demographics, tumor characteristics, diagnosis, initial treatment regimen, and vital status updates. The SEER database strictly maintains patient confidentiality and does not disclose personally identifiable information. Therefore, relevant analyses of SEER data are not subject to medical ethical review or the need to obtain informed consent from participants. The study also collected clinicopathologic data from external medical institutions (Wenzhou, China). In 2015, immunotherapy-related drugs were officially approved for the treatment of patients with advanced lung cancer. This approval not only means the resolution of the treatment stalemate for advanced lung cancer patients but also heralds the official entry of lung cancer treatment into the immunization era. To assess the effectiveness of immunotherapy, we specifically targeted patients diagnosed with bone metastases from lung adenocarcinoma in 2015-2020, consistent with the approval of immunotherapy as a primary treatment modality in 2015. In contrast, patients with bone metastases from lung adenocarcinoma diagnosed between 2010-2015 were the comparison cohort for the study. Inclusion criteria were as follows: 1. Lung cancer was the only primary tumor; 2 Lung adenocarcinoma was diagnosed by histological examination. Exclusion criteria were: 1. concomitant multiple primary tumors; 2. incomplete information on relevant tumor characteristics; and 3. incomplete information on treatment and follow-up. Variables such as age, race, sex, primary tumor site, laterality, surgery (no or yes), radiotherapy (no or yes), chemotherapy (no or yes), immunotherapy (no or yes), lung metastasis (no or yes), liver metastasis (no or yes), brain metastasis (no or yes), and marital status were included in this study. The histological type was categorized into adenocarcinoma, squamous cell carcinoma, small cell lung cancer, and others based on pathologic findings. The optimal age threshold under OS was determined to be 74 using X-tile software, so age was categorized as <74 years and ≥74 years. The primary endpoint in this study was OS, which was defined as the date from diagnosis to death or last follow-up.

### Statistical analysis

Selection bias inevitably permeated this study due to inconsistencies in the baseline characteristics of patients in the group receiving immunotherapy and those in the group not receiving immunotherapy. To reduce the impact of differences in baseline characteristics on OS, this study used a 1:1 propensity score matching (PSM method, setting a caliper width of 0.01 to harmonize between patients in the group receiving immunotherapy and those in the group not receiving immunotherapy. Subsequently, Kaplan-Meier curve analysis and log-rank test were performed to measure the effect of immunotherapy on OS of LABM. Data from the total SEER cohort were randomly divided into a training cohort and an internal validation cohort in a 7:3 ratio using R software. The training cohort was used to develop the model, the internal validation cohort was used for internal validation of the model, and the collected external validation cohort performed external validation. Univariate and multivariate analyses were performed in the training cohort using Cox regression risk models. Variables significantly associated with survival in the univariate analysis were then included in the multivariate Cox analysis to exclude confounding effects between variables. Clinical predictive models based on independent prognostic factors were constructed using R software and validated and evaluated in three cohorts. The area under the receiver operating characteristic curve was used to assess the discriminatory nature of the model, and the calibration curve was used to assess the predictive accuracy of the model. Decision curves were used to assess the predictive performance and clinical benefits of the models. A risk classification system was developed based on the clinical prediction model to successfully differentiate LABM at high, middle, and low risk of death. To determine the maximum beneficiary population of immunotherapy in each death risk subgroup, the study further conducted a subgroup analysis of each death risk group using Kaplan-Meier curve analysis and log-rank test. All statistical analyses in this study were performed using R software (version 4.3.3), where p<0.05 was considered statistically significant.

## Results

### Demographic and clinicopathologic features

In this study, we performed a comprehensive analysis of the baseline demographic and clinical characteristics of the cohort involved (**Table 1**). A total of 14051 individuals were included in the training cohort, and there were no missing age or sex data in this group. Similarly, the internal validation cohort included 6022 subjects and showed no missing values for these demographics. Age distribution within the cohort showed no significant difference between the training cohort (73.5% <74 years and 26.5% ≥74 years) and the internal validation cohort (73.0% <74 years and 27.0% ≥74 years), with a P-value of 0.522. In terms of racial composition, the difference between the two cohorts was nonsignificant, with a majority of the cohort in both groups being white. Gender distribution analysis showed a similar pattern, with no significant differences found (P-value= 0.859). The primary site of the tumor did not differ between the two groups, with the majority of cases located in the upper lobes of the lungs (59.5% in the training cohort and 60.2% in the internal validation cohort). There were no statistically significant differences between groups for laterality, immunotherapy, surgery, radiotherapy, chemotherapy, brain metastasis, liver metastasis, lung metastasis, and marital status, with p-values ranging from 0.076 to 0.974. Together, these findings demonstrate a high degree of concordance between the training cohort and internal validation cohort in terms of demographic and clinical characteristics, providing a solid foundation for subsequent predictive modeling studies.

**Table 1:** Demographic and clinicopathological characteristics of training cohort and internal validation cohort.

Characteristic	Cohort		
	Training Cohort N = 14051	Internal validation Cohort N = 6022	P-value
**Age**			0.522
<74	10323 (73.5%)	4398 (73.0%)	
≥74	3728 (26.5%)	1624 (27.0%)	
**Race**			0.091
Black	1468 (10.4%)	569 (9.4%)	
White	10629 (75.6%)	4593 (76.3%)	
Others	1954 (13.9%)	860 (14.3%)	
**Sex**			0.859
Male	7250 (51.6%)	3099 (51.5%)	
Female	6801 (48.4%)	2923 (48.5%)	
**Primary site**			0.487
Main bronchus	548 (3.9%)	237 (3.9%)	
Upper lobe	8354 (59.5%)	3627 (60.2%)	
Middle lobe	675 (4.8%)	304 (5.0%)	
Lower lobe	4474 (31.8%)	1854 (30.8%)	
**Laterality**			0.974
Left	6028 (42.9%)	2585 (42.9%)	
Right	8023 (57.1%)	3437 (57.1%)	
**Immunotherapy**			0.530
No	5587 (39.8%)	2423 (40.2%)	
Yes	8464 (60.2%)	3599 (59.8%)	
**Surgery**			0.558
No	13789 (98.1%)	5917 (98.3%)	
Yes	262 (1.9%)	105 (1.7%)	
**Radiotherapy**			0.076
No	5705 (40.6%)	2526 (41.9%)	
Yes	8346 (59.4%)	3496 (58.1%)	
**Chemotherapy**			0.526
No	4944 (35.2%)	2147 (35.7%)	
Yes	9107 (64.8%)	3875 (64.3%)	
**Brain metastasis**			0.845
No	9799 (69.7%)	4208 (69.9%)	
Yes	4252 (30.3%)	1814 (30.1%)	
**Liver metastasis**			0.164
No	10816 (77.0%)	4581 (76.1%)	
Yes	3235 (23.0%)	1441 (23.9%)	
**Lung metastasis**			0.278
No	10090 (71.8%)	4279 (71.1%)	
Yes	3,961 (28.2%)	1743 (28.9%)	
**Marital status**			0.017
No	5804 (41.3%)	2597 (43.1%)	
Yes	8247 (58.7%)	3425 (56.9%)	

### Selection of study cohort and propensity score matching

To reduce the effect of confounding variables, a 1:1 PSM strategy was used to produce a final matched cohort consisting of 12063 cases in the ‘received immunotherapy’ group and 8010 cases in the ‘did not receive immunotherapy’ group. Following this matching process, the two cohorts exhibited a high degree of concordance in baseline characteristics, as shown in **Table 2**. This improved balance makes comparisons of treatment effects more reliable because the potential effects of confounders are minimized. The observed increase in p-values for previously significant covariates demonstrates the effectiveness of our matching technique in addressing potential confounders. This enhances the credibility of our findings and applies them to real clinical practice.

**Table 2:** Baseline characteristics of patients before and after propensity score matching.

Characteristics	Unmatched			Matched		
	Yes, N = 12063	No, N = 8010	p-value	Yes, N = 7924	No, N = 7924	p-value
**Age**			<0.05			0.73
<74	8728 (72%)	5993 (75%)		5942 (75%)	5923 (75%)	
≥74	3335 (28%)	2017 (25%)		1982 (25%)	2001 (25%)	
**Race**			<0.05			0.51
Black	1221 (10%)	816 (10%)		770 (10%)	807 (10%)	
White	9018 (75%)	6,204 (77%)		6194 (78%)	6137 (77%)	
Others	1824 (15%)	990 (12%)		960 (12%)	980 (12%)	
**Sex**			<0.05			0.83
Male	6113 (51%)	4236 (53%)		4192 (53%)	4179 (53%)	
Female	5950 (49%)	3774 (47%)		3732 (47%)	3745 (47%)	
**Primary site**			<0.05			0.24
Main bronchus	455 (4%)	330 (4%)		280 (4%)	322 (4%)	
Upper lobe	7168 (59%)	4813 (60%)		4828 (61%)	4775 (60%)	
Middle lobe	558 (5%)	421 (5%)		372 (5%)	399 (5%)	
Lower lobe	3882 (32%)	2446 (31%)		2444 (31%)	2428 (31%)	
**Laterality**			0.560			0.48
Left	5156 (43%)	3457 (43%)		3376 (43%)	3420 (43%)	
Right	6907 (57%)	4553 (57%)		4548 (57%)	4504 (57%)	
**Surgery**			<0.05			0.65
No	11866 (98%)	7840 (98%)		7805 (98%)	7798 (98%)	
Yes	197 (2%)	170 (2%)		119 (2%)	126 (2%)	
**Radiotherapy**			<0.05			0.60
No	5039 (42%)	3192 (40%)		3131 (40%)	3163 (40%)	
Yes	7024 (58%)	4818 (60%)		4793 (60%)	4761 (60%)	
**Chemotherapy**			<0.05			0.86
No	4377 (36%)	2714 (34%)		2676 (34%)	2686 (34%)	
Yes	7686 (64%)	5296 (66%)		5248 (66%)	5238 (66%)	
**Brain metastasis**			<0.05			0.88
No	8232 (68%)	5775 (72%)		5692 (72%)	5700 (72%)	
Yes	3831 (32%)	2235 (28%)		2232 (28%)	2224 (28%)	
**Liver metastasis**			<0.05			0.70
No	9187 (76%)	6210 (78%)		6165 (78%)	6145 (78%)	
Yes	2876 (24%)	1800 (22%)		1759 (22%)	1779 (22%)	
**Lung metastasis**			0.526			0.47
No	8655 (72%)	5714 (71%)		5715 (72%)	5674 (72%)	
Yes	3408 (28%)	2296 (29%)		2209 (28%)	2250 (28%)	
**Marital status**			0.250			0.82
No	5088 (42%)	3313 (41%)		3265 (41%)	3279 (41%)	
Yes	6975 (58%)	4697 (59%)		4659 (59%)	4645 (59%)	

### Immunotherapy and survival outcomes

**Figure 1:**
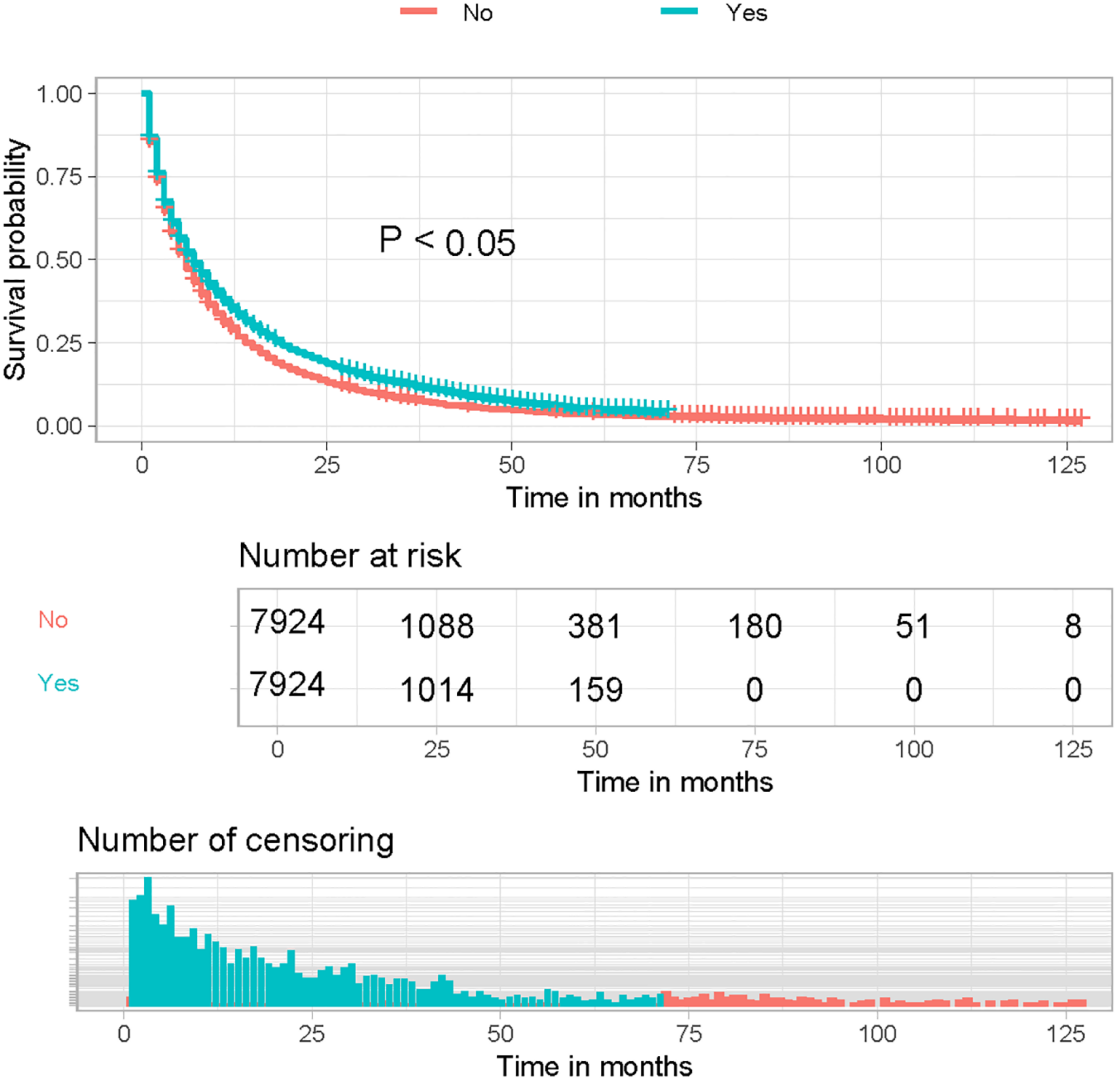
Kaplan-Meier curves for immunotherapy of patients after propensity score matching.

Kaplan-Meier curves of the post-PSM cohort showed a significant improvement in OS in LABM who received immunotherapy compared to those who did not (**Figure 1**, P < 0.05). These results suggest that immunotherapy can significantly improve the prognosis of LABM.

### Construction and validation of the model

**Figure 2:**
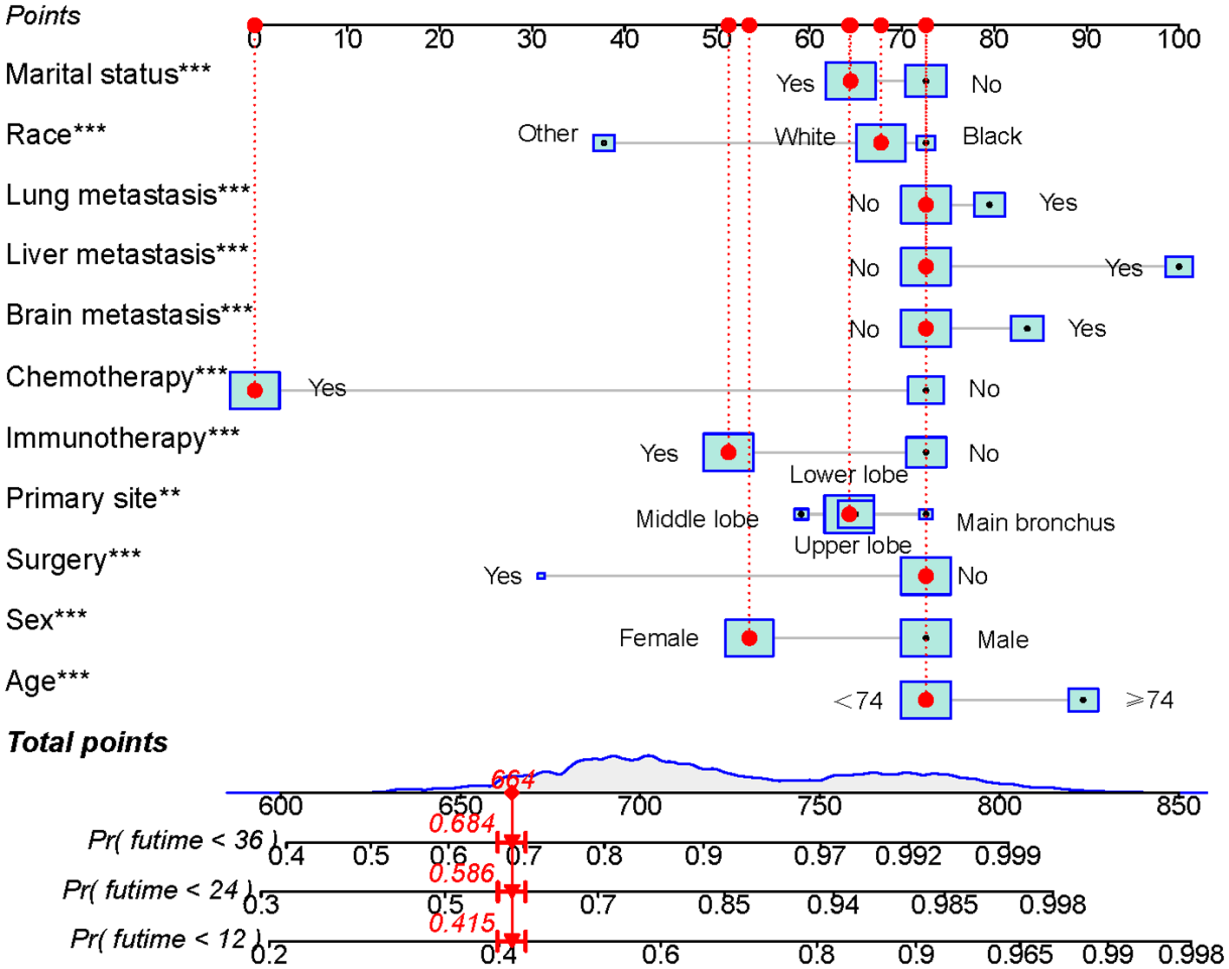
Prognostic model for predicting 1-, 2-, and 3-year OS probability in LABM. Symbol ** represent p < 0.01 and symbol *** represent p < 0.001.

**Figure 3:**
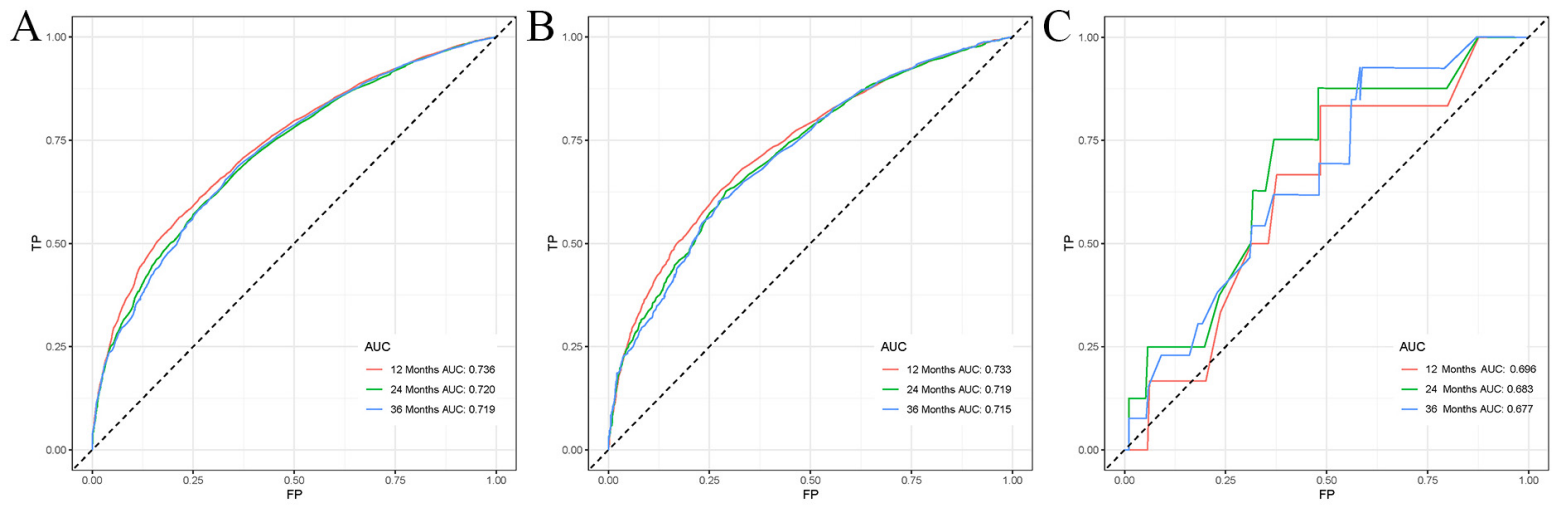
ROC curves for LABM. **(A)** ROC curves of 1-, 2-, and 3 years in the training cohort, **(B)** ROC curves of 1-, 2-, and 3 years in the internal validation cohort, **(C)** ROC curves of 1-, 2-, and 3-year in the external validation cohort.

**Figure 4:**
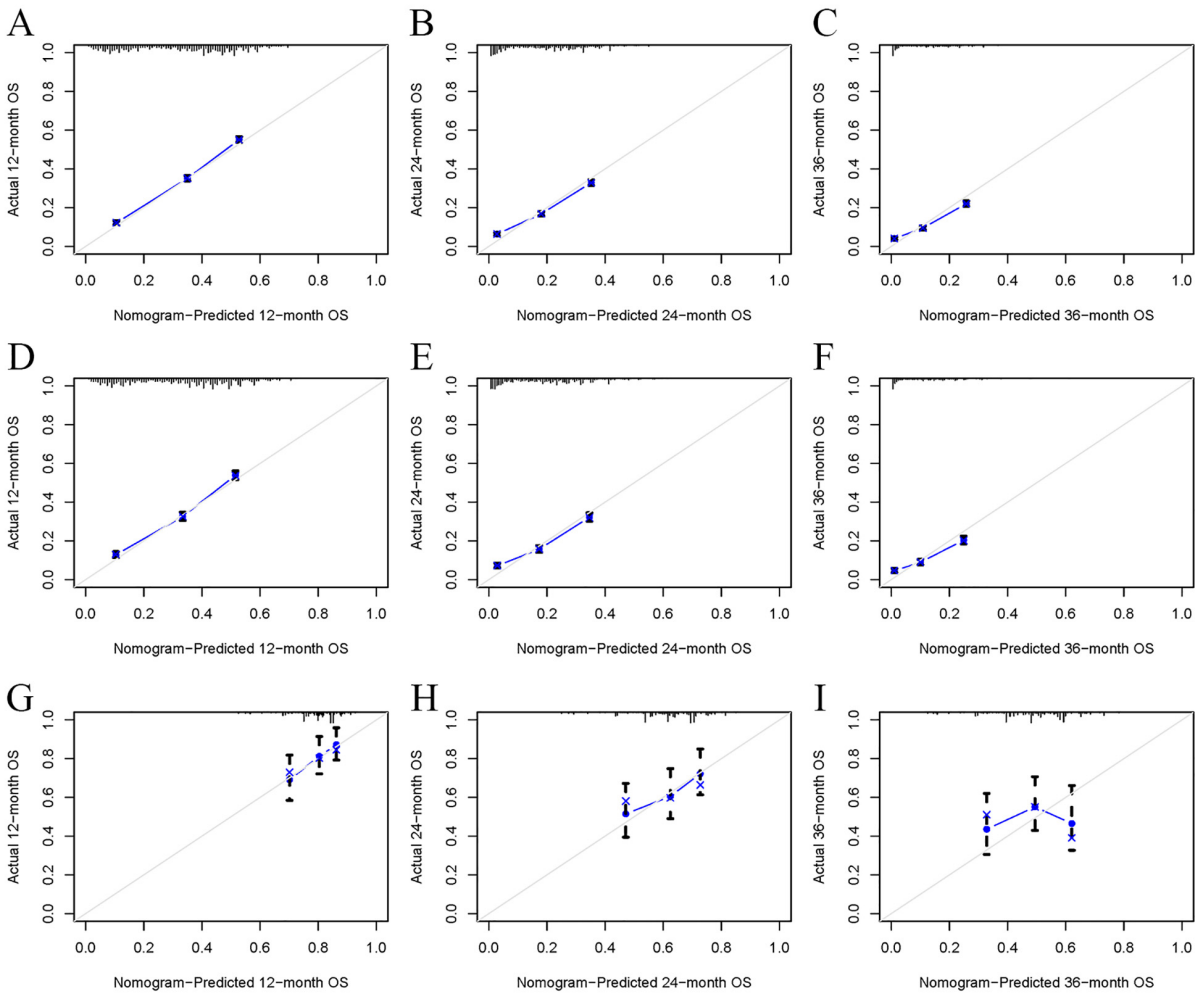
The calibration curves of the model for the prediction of the OS of patients in the training cohort **(A–C)**, internal validation cohort **(D–F)**, and external validation cohort **(G–I)**.

**Figure 5:**
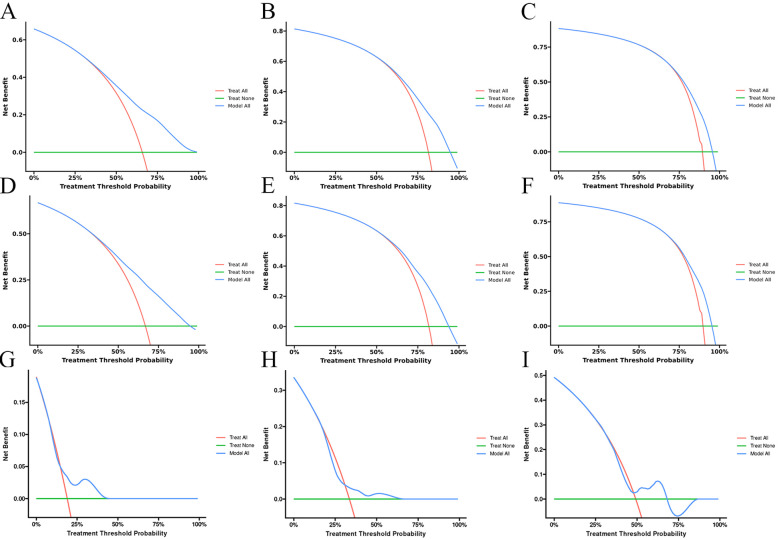
The decision curves of the model for the prediction of the OS in the training cohort **(A–C)**, internal validation cohort **(D–F)**, and external validation cohort **(G–I)**.

Univariate Cox regression analysis revealed that age, race, sex, primary tumor site, surgery, chemotherapy, immunotherapy, lung metastasis, liver metastasis, brain metastasis, and marital status were significantly associated with the prognosis of LABM (P < 0.05, **Table 3**). To remove the confounding effects among variables, the above-screened risk factors were further analyzed by multivariate Cox regression analysis. Multivariate Cox regression analysis showed that age, race, sex, primary tumor site, surgery, chemotherapy, immunotherapy, lung metastasis, liver metastasis, brain metastasis, and marital status were independent prognostic factors in LABM (P < 0.05, **Table 3**). We developed a clinical prediction model based on the screened independent prognostic factors to achieve accurate prediction of 1-, 2-, and 3-year OS in LABM. Each variable of a patient corresponds to a score value, and the corresponding score values are summed to obtain a total score, which gives the patient’s survival probability at 1, 2, and 3 years (**Figure 2**). The performance of the clinical prediction model was validated in both the training cohort and the internal validation cohort as well as the external validation cohort. According to the ROC curve analysis, the time-dependent AUC values for 1, 2, and 3 years were 0.736, 0.720, and 0.719 for the training cohort, 0.733, 0.719, and 0.715 for the internal validation cohort, and 0.696, 0.683, and 0.677 for the external validation cohort, respectively (**Figure 3**). These results confirm that the model has good discriminatory power. The calibration curves for survival probability showed that the model had the best correlation between predictions and observations of OS in all three cohorts, further confirming that the model developed in this study reliably predicted patient survival (**Figure 4**). Decision curve analysis, on the other hand, demonstrated the significant positive net benefit of the model over a wide range of mortality risks, further proving the high clinical utility of the model for LABM (**Figure 5**).

**Table 3:** Analysis of univariate and multivariate Cox regression in patients.

Variables	Univariate analysis		Multivariate analysis	
	HR (95% CI)	P value	HR (95% CI)	P value
Age				
<74	Reference		Reference	
≥74	1.41 (1.35-1.46)	<0.05	1.25 (1.20-1.30)	<0.05
Sex				
Male	Reference		Reference	
Female	0.80 (0.77-0.83)	<0.05	0.78 (0.75-0.81)	<0.05
Race				
Black	Reference		Reference	
White	0.91 (0.86-0.96)	<0.05	0.94 (0.88-1.00)	<0.05
Others	0.59 (0.55-0.64)	<0.05	0.64 (0.59-0.69)	<0.05
Laterality				
Left	Reference			
Right	1.03 (1.00-1.07)	0.09		
Primary site				
Main bronchus	Reference		Reference	
Upper lobe	0.86 (0.79-0.95)	<0.05	0.90 (0.82-0.99)	<0.05
Middle lobe	0.82 (0.72-0.92)	<0.05	0.84 (0.74-0.95)	<0.05
Lower lobe	0.85 (0.77-0.94)	<0.05	0.91 (0.82-1.00)	<0.05
Immunotherapy				
No	Reference		Reference	
Yes	0.80 (0.77-0.83)	<0.05	0.76 (0.73-0.79)	<0.05
Radiotherapy				
No	Reference			
Yes	1.00 (0.97-1.04)	0.82		
Chemotherapy				
No	Reference		Reference	
Yes	0.38 (0.37-0.40)	<0.05	0.39 (0.38-0.41)	<0.05
Surgery				
No	Reference		Reference	
Yes	0.60 (0.53-0.69)	<0.05	0.58 (0.51-0.67)	<0.05
Brain metastasis				
No	Reference		Reference	
Yes	1.04 (1.00-1.08)	<0.05	1.15 (1.11-1.20)	<0.05
Liver metastasis				
No	Reference		Reference	
Yes	1.36 (1.31-1.42)	<0.05	1.43 (1.37-1.49)	<0.05
Lung metastasis				
No	Reference		Reference	
Yes	1.11 (1.06-1.15)	<0.05	1.09 (1.05-1.14)	<0.05
Marital status				
Unmarried	Reference		Reference	
Married	0.82 (0.79-0.85)	<0.05	0.90 (0.87-0.93)	<0.05

### Risk classification system

**Figure 6:**
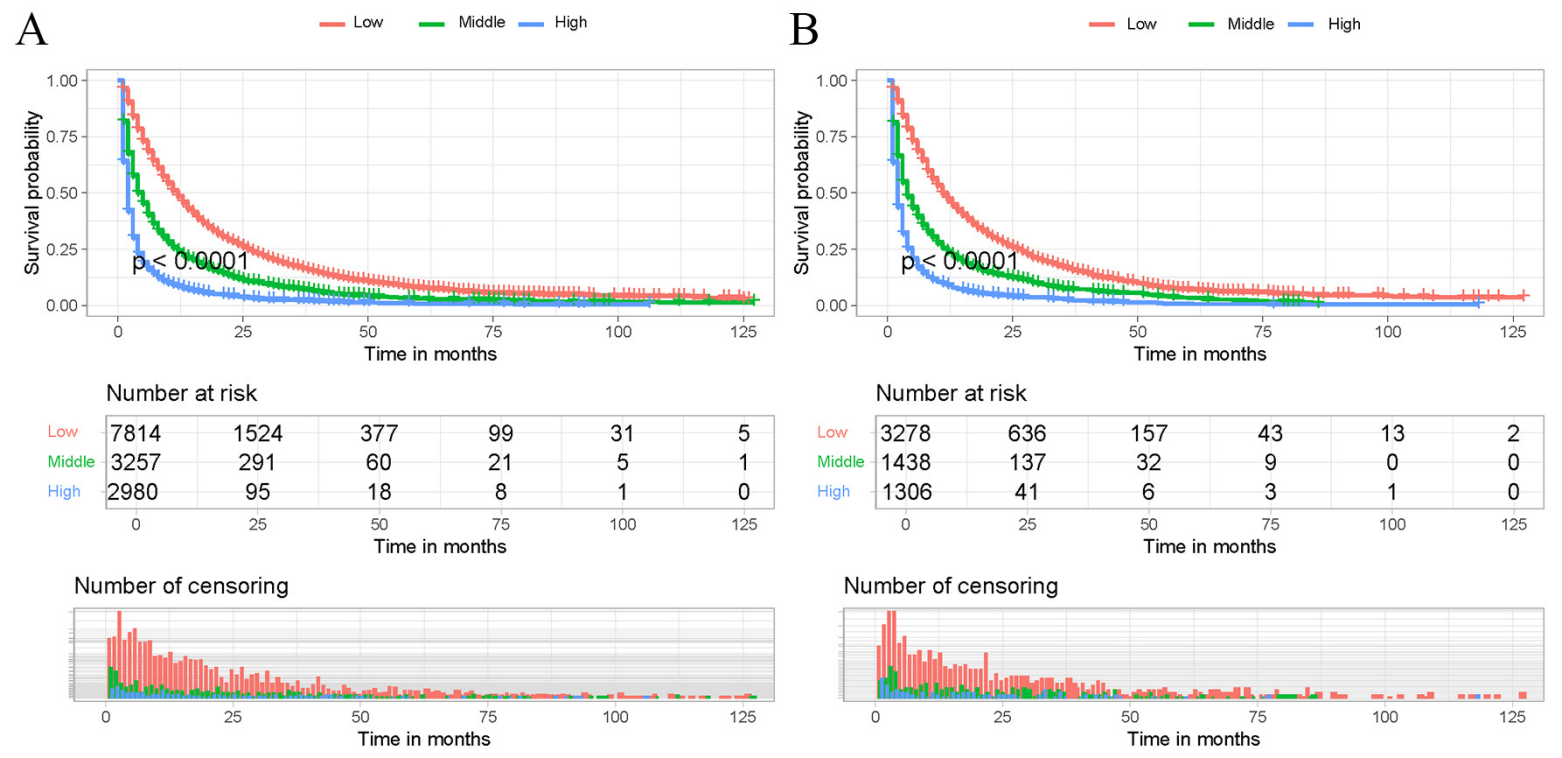
Kaplan-Meier survival curves of the training cohort and internal validation cohort. Patients in the training cohort **(A)** and internal validation cohort **(B)** with a higher risk score demonstrated worse OS than those with a lower risk score.

Based on the developed predictive model to calculate the total score for each patient in the training cohort, the optimal cut-off values of 724 and 770 were determined using X-tiles software. Thus, all patients were categorized into low-risk risk group (scores < 724), middle-risk group (724–770), and high-risk risk group (scores > 770). Kaplan-Meier survival curves for each risk subgroup were further plotted and log-rank tests were performed. The results, as shown in **Figure 6**, showed that there was a significant difference in the prognosis of patients in different risk groups in the training and internal validation cohorts (p<0.05). The above results indicate that the risk classification system derived from the clinical prediction model has a high prognostic predictive value and can further distinguish the population with a better prognosis.

### Determining the optimal beneficiary population for immunotherapy based on the risk classification system

**Figure 7:**
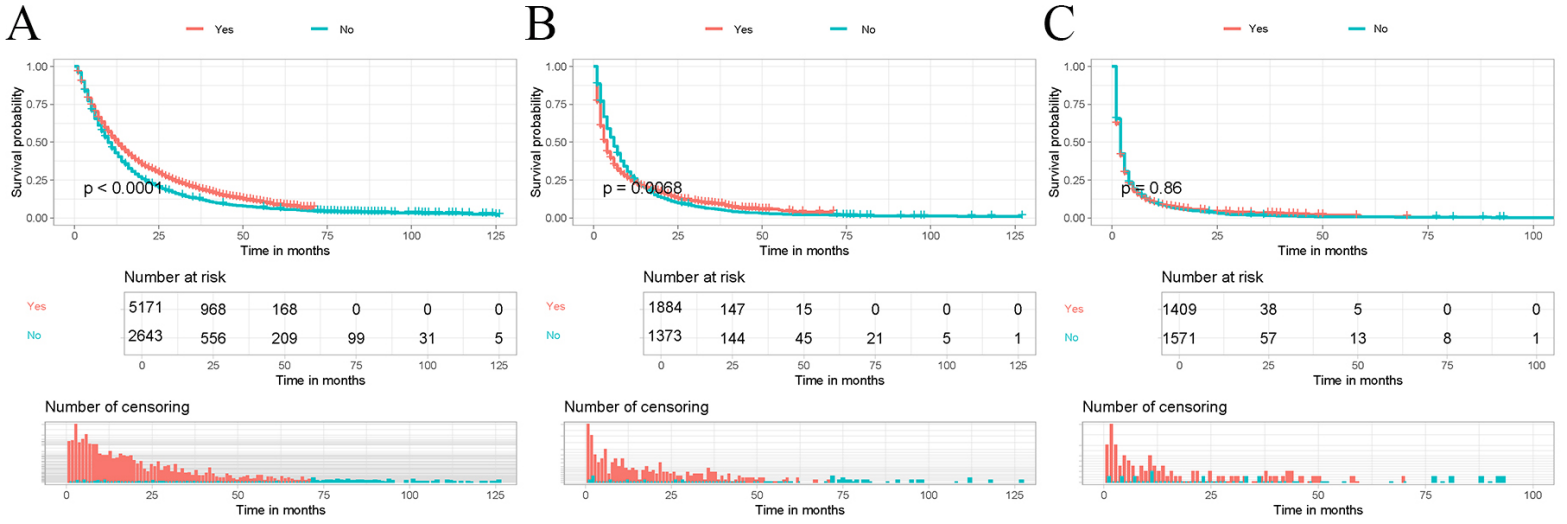
Kaplan-Meier curves were performed to analyze the OS of patients in different mortality risk groups by immunotherapy. **(A)** Kaplan-Meier curve for immunotherapy in the low-risk group; **(B)** Kaplan-Meier curve for immunotherapy in the middle-risk group; **(C)** Kaplan-Meier curve for immunotherapy in the high-risk group.

The correlation of immunotherapy was analyzed by depicting Kaplan-Meier survival curves and performing log-rank tests for different risk subgroups differentiated by a risk classification system. As shown in **Figure 7**, the OS of patients who received immunotherapy in the high-risk and middle-risk groups was not significantly different from that of patients who did not receive immunotherapy. In contrast, the OS of patients who received immunotherapy in the low-risk risk group was significantly better than that of patients who did not receive immunotherapy (P < 0.05). Therefore, we prefer to recommend immunotherapy to LABM who are distinguished as low-risk by the risk classification system because they can benefit most from immunotherapy.

## Discussion

The treatment of lung cancer has fundamentally changed over the past two decades, especially with the intensive development of molecular pathology of lung cancer and the rise of immunotherapy. Pembrolizumab achieved a 5-year OS rate of 31.9% in the prestigious Keynote-024 trial, which was approved by the FDA as an effective first-line therapeutic option for patients with non-small cell lung cancer (12). However, it is important to recognize that not all patients will benefit from immunotherapy. Considering the poor prognosis of LABM, the therapeutic efficacy of immunotherapy in these patients is unknown, and there are fewer previous related reports. Therefore, this study retrospectively analyzed the data from the multicenter SEER database, firstly verified the significant prognostic improvement of immunotherapy in LABM by PSM, and then constructed a model for predicting the survival of LABM. The performance of the model was also externally validated by collecting data from relevant patients from external medical institutions who met the inclusion-exclusion criteria. Finally, a risk classification system was constructed based on the predictive model, by which we successfully screened the population that could benefit most from immunotherapy among LABM. This provides strong evidence for rational allocation of medical resources and personalized and precise treatment.

Our study demonstrated that LABM who received immunotherapy had significantly longer OS than those who did not receive immunotherapy, similar to previous studies. However, the current study included a larger number of patients compared to the previous study, and the patients were from multiple central medical institutions. Therefore, the results of this study are more compelling and representative. In this study, female patients were predictors of good prognosis, which is similar to previously reported results. Smoking rates are lower in women than in men, and therefore nonsmoking-related lung cancers (e.g., lung adenocarcinoma) are more prevalent in women (15). These types of lung cancers usually have milder biological behavior and are more sensitive to specific treatments (16). In addition, it has been proposed that sex hormones, particularly estrogen, may influence the biological behavior and response to treatment in lung cancer. Estrogen receptors are expressed in certain lung adenocarcinoma cells, which may influence how tumors grow and spread (17). Gender has been reported to affect innate and acquired immune responses, as well as the expression and function of PD-L1 and PD-1 (18). Some studies suggest that women may be more resistant to immunotherapy because their tumor immunogenic response is weaker than men’s (19). Some studies report that immunotherapy is more effective for men than women in non-small cell lung cancer (20). Bone metastases play a key role in modulating the immune response and influencing the response to immunotherapy (21, 22). Thus, among LABM female patients may have a better response to immunotherapy and thus have a better prognosis compared to male patients.

Bone metastases have traditionally been treated without cure, with patients receiving multidisciplinary treatment based on systemic therapy and optimal local therapy, including radiotherapy, targeted therapy, immunotherapy, and surgery (23).In these treatments, instead of targeting the primary site, surgery removes isolated bone metastases. The aim is to prevent and treat pathologic fractures and to reduce bone pain and spinal cord compression to improve the patient’s quality of life (24). However, with advances in surgical techniques and multidisciplinary approaches to care, adverse events associated with surgical death have decreased, and resection of the primary tumor has been reconsidered as part of the treatment of advanced lung adenocarcinoma (25).In recent years, several studies have shown that resection of the primary tumor improves the prognosis of stage IV non-small cell lung cancer (26, 27). Although there are no guidelines for re-recommending this therapy for patients with bone metastases from advanced non-small cell lung cancer, some evidence supports surgical treatment for them (28). This study suggests that surgery is an independent prognostic factor for LABM. In our study, we focused on a more specific type of lung cancer with bone metastases because it helps to make more precise individualized decisions and because lung adenocarcinoma has the highest incidence of bone metastases. We believe that removing the primary tumor slows tumor progression by reducing tumor load and decreasing the release of tumor cells into the bloodstream (29). Of course, not all LABM are suitable for surgical treatment by resection of the primary tumor, and more research is still needed in the future to further explore which LABM are more suitable for surgical treatment.

We found that chemotherapy is an independent prognostic risk factor for LABM. Chemotherapy drugs can shrink tumors and control their growth and spread by destroying rapidly dividing cancer cells (30). This can provide some relief of symptoms, pain, and other complications caused by the tumor in patients who have developed bone metastases. In some cases, chemotherapy may be the initial treatment option, especially if the patient has low levels of PD-L1 expression or no targetable gene mutations (31). Chemotherapy may be used as a first-line treatment to stabilize the disease and provide an opportunity for subsequent treatment. Confusingly, the study suggests that radiotherapy is not a prognostic factor for LABM. Although radiotherapy is not usually a curative treatment, it can be effective in controlling the local spread of tumors. It offers a relatively gentle form of management for patients whose health or age precludes them from receiving more aggressive treatments such as chemotherapy or major surgery (32). We believe that for patients with bone metastases, radiotherapy is only a local treatment for relief of bone pain and prevention of pathologic fractures, and does not slow the progression of the tumor in a way that serves to prolong patient survival (33). Secondly, high doses and long courses of radiotherapy may cause severe myelosuppression, leading to a worse prognosis for the patient.

Although immunotherapies have shown significant efficacy in the treatment of lung cancer, to date, no large multicenter cohort study has analyzed their potential efficacy in LABM. It is important to emphasize that even though studies have shown that immunotherapy can improve survival in LABM, it has been shown that not all patients can benefit from it. Therefore, a risk classification system was further developed to better allocate healthcare resources and to enable precision treatment. By using the risk classification system to distinguish those who can benefit most from immunotherapy, we can achieve personalized and precise treatment, avoiding waste of resources and over-treatment of patients. However, the study has some limitations. First, as a multicenter retrospective study, selection bias is inevitable. Second, the patient-related information available in the SEER database is limited. For example, laboratory test results and relevant imaging results were missing. In the future, further large-scale prospective multicenter studies are needed to develop a more comprehensive and accurate risk classification system to differentiate the optimal beneficiary population for immunotherapy in LABM.

## Conclusions

This study first demonstrated that immunotherapy can significantly improve the prognosis of LABM through a multicenter large-scale retrospective study. Second, a risk classification system was constructed to screen the best beneficiary population for immunotherapy in LABM. This tool can be extremely useful in the field of clinical decision-making, laying the foundation for personalized and precise treatment, thus avoiding the waste of medical resources and over-treatment.

## Data Availability

The original contributions presented in the study are included in the article/supplementary material, further inquiries can be directed to the corresponding author/s.
